# In the liminal spaces of mental health law – what to do when section 136 expires?

**DOI:** 10.1192/bjb.2022.59

**Published:** 2023-12

**Authors:** Khalil Hassanally, Judy Laing, Anupam Kishore

**Affiliations:** 1 Northwick Park Hospital, Central and North West London NHS Foundation Trust, London, UK; 2 University of Bristol Law School, Bristol, UK; 3 Park Royal Centre for Mental Health, Central and North West London NHS Foundation Trust, London, UK

**Keywords:** Psychiatry and law, consent and capacity, education and training, ethics, human rights

## Abstract

The pressure on mental health services has not gone unremarked and is of widespread concern in England and Wales. This can have implications when a bed is being sought for a patient who has undergone a Mental Health Act assessment and is deemed to meet the criteria for being formally admitted to hospital. Once the 24 h period for assessment under section 136 of the Act has lapsed, the ongoing detention of the patient can lead to a legal grey area. Through a fictional example this paper examines the relevant case law and statute that may be used to continue the detention and explores the ethical problems that this may cause.

## Clinical scenario

It is early on Friday evening, and a 35-year-old man has been brought to a health-based place of safety (HBPoS) under section 136 of the Mental Heath Act by police officers. They had initially detained him after he had run into the road holding a large knife, claiming he was being chased by spirits who wish to do him harm. He has had one previous brief contact with mental health services when his brother brought him to a hospital accident and emergency (A&E) department. At that time, it was felt that he may be suffering from psychotic symptoms, but the presentation was complicated by the fact that he may have also been misusing illicit substances. It was thought he could be managed by community services, but he did not follow up and has had no further contact. As the higher trainee on call, you attended for a Mental Health Act assessment, and along with the independent section 12 doctor and the approved mental health professional (AMHP) you felt that detention was warranted because he posed a risk to his own health and safety, as well as the safety of others were he to be released. None of you felt he had the capacity to make decisions about admission or treatment. The following day, while still on call you are called by the HBPoS staff as the period for his section 136 detention is coming to an end and there are no beds available and there are unlikely to be any this weekend. After further review it is felt that there has been little change in his presentation and that there are still risks to both himself and the safety of others. The staff are unsure what to do when the detention period comes to an end and ask you for your advice:

- What are the immediate next steps you should take?
- Where can you turn for emergency legal advice over the weekend?
- Under what legal authority could you continue to hold the patient?

## Discussion

### Review of the law

The 2017 amendments to the Mental Health Act 1983 (MHA) reduced the time that a patient could spend in detention in a place of safety under section 136 of the Act from 72 h to 24 h.^1^ There remains an option to extend the assessment period for a further 12 h, but only in limited circumstances where the patient's condition is such that it would not be practicable for the assessment to be completed before the expiry of the initial period.^1,^^2^ The fictitious scenario described above is becoming common in some areas of England and Wales owing to an increased use of section 136 by the police,^3^ coupled with an ongoing reduction in psychiatric beds.^4^ Ideally, following a section 136 MHA assessment, the patient should be admitted informally, or detained under one of the civil sections of the MHA or the section 136 should be rescinded. In the situation described, where the patient requires admission under another section but there are no beds available and the section 136 has lapsed, the MHA and its Code of Practice do not provide a solution as to what should be done.

In the given scenario all the assessors agree that the patient lacks the capacity to make a decision as to admission or treatment. This gives rise to consideration of whether the Mental Capacity Act 2005 (MCA) can be used. Although the interface and differences between the MHA and MCA are complex, for our purposes it is important to note that they have different emphases and are not interchangeable.^5^ The MCA makes the patient's capacity to make a decision its primary concern, whereas the MHA has a stronger emphasis on risk.^5,6^ There will be occasions when a patient may be subject to both the MCA and the MHA, for example a patient detained in a psychiatric hospital who lacks capacity to make a decision about their physical health,^6^ but in the scenario we are facing this does not apply as the focus is on the person's mental disorder. As per paragraph 5 of Schedule 1A of the MCA,^1,4^ a patient who would fall within the scope of the MHA cannot be detained under the MCA if they would object to mental health treatment.^6,7^ This point was further made in the case of *DN v Northumberland, Tyne and Wear NHS Foundation Trust* [2011], where (though not party to the case) the Department of Health and Social Care had provided analysis (a policy rather than legal position) that:

‘[i]n broad terms (and subject to certain caveats), it means that the MCA cannot be used to deprive someone of their liberty in a hospital for the purposes of mental health treatment if they are objecting to that course of action and they could instead be detained under the MHA’.^8^

Furthermore, the intention of the policy was that:

‘people who lack capacity to consent to being admitted to hospital, but who are clearly objecting to it, should generally be treated like people who have capacity and are refusing to consent to mental health treatment. If it is considered necessary to detain them in hospital, and they would have been detained under the MHA if they had the capacity to refuse treatment, then as a matter of policy it was thought right that the MHA should be used in preference to the MCA’.^8^

Therefore, in the scenario given, the Department of Health's policy advice suggests that the MCA should not be used to extend the person's detention. Logically, this should also preclude us from using the Deprivation of Liberty Safeguards (DoLS) introduced as an amendment to the MCA. It is worth noting that the DoLS regime is being replaced with the Liberty Protection Safeguards (LPS, introduced in the Mental Capacity (Amendment) Act 2019) as further amendments to the MCA. Within the amended MCA (which is not yet in force), section 4B references when a patient can be deprived of their liberty in an emergency. The four conditions needed prior to being able to utilise these powers are that:

‘1. the steps consist of, or are for purpose of, giving a life-sustaining treatment or carrying out a vital act

2. the steps are necessary in order to give the life-sustaining treatment or carry out the vital act

3. the decision-maker believes that the person lacks capacity to consent to the steps taken

4. a relevant decision is being sought from the court, a Responsible Body is determining whether to authorise arrangements under the LPS, or there is an emergency’.^9^

In the circumstances described it would be difficult to argue that the use of these powers would constitute a ‘vital act’, examples of which are given in a new draft Code of Practice for the MCA, currently under consultation.^9^ Furthermore, there has been nothing so far to suggest that the Department of Health is looking to change its policy on the use of the MCA in this area. The recently released draft Mental Health Bill does not make any mention of changes to the period that a patient may be detained under section 136 and looks to abolish the use of police custody as a place of safety, which may potentially further increase the pressure on mental health services, thereby exacerbating the situation.^10^

There is no broad common law (also known as case or judge-made law) authority to deprive a person of their liberty in this situation either. Although neither the MHA nor the MCA specifically abrogate common law powers,^11^ in the case of *R (Sessay) v South London and Maudsley NHS Foundation Trust* [2011] (which concerned a patient refusing admission and who was felt to lack capacity), Mr Justice Supperstone held that ‘[t]here is no power to deprive patients, such as the Claimant, of their liberty in psychiatric hospitals under the common law doctrine of necessity’,^12^ further stating that ‘Part II of the Mental Health Act 1983 provides a comprehensive code for compulsory admission to hospital for non-compliant incapacitated patients’.^12^ This seems to preclude any reliance on the common law defence of necessity to extend detention in this instance.^6^

Holding a patient without proper legal authority means that they might be falsely imprisoned or unlawfully deprived of their liberty.^13,14^ It is worth pointing out that false imprisonment and unlawful deprivation of liberty are not the same: although all cases of false imprisonment will result in an unlawful deprivation of liberty the converse is not always true.

False imprisonment is an offence at common law; the basis for false imprisonment was set out in the case of *R v Rahman* (1985), where it was held that the prosecution needs to prove the detention is:^15^

- unlawful; and
- intentional or reckless; and
- a restraint of a victim's freedom of movement from a particular place.

Restraint can be physical, for example the locking of a patient in a room, as in the case of *R v Linsberg and Leies* (1905),^15^ or non-physical, for example by intimidation or threats of legal action, as in the case of *R v Anthony David James* (1997) – where although the defendant could have physically left the room, he was scared to do so.^15^

An unlawful deprivation of liberty would be a breach of the person's fundamental human rights under Article 5 of the European Convention on Human Rights.^13,14^ In the case of *R (Jalloh) v Secretary of State for the Home Department* [2020] (an immigration case that pertains to detention) the court held that, when deciding on unlawful deprivation of liberty, it was important ‘to look at the restraint in question in the context of the whole picture: and a distinction between deprivation of liberty on the one hand and restriction on movement on the other hand is maintained, involving an assessment of the whole range of factors present, including nature, duration and effects of the restraint, the manner of implementation and execution and so on’.^13^ Depending on the circumstances and nature of the detention or restraint, once the section 136 has lapsed, it may be that the person is falsely imprisoned or unlawfully deprived of their liberty.

There is provision that in an emergency, when urgent life-saving treatment is necessary owing to an imminent risk to life, if the patient is not be able to consent and treatment would require restriction of their liberty (for example treating an unconscious person in an intensive care unit), it would not amount to an unlawful deprivation of their liberty.^6,16^ However, similar to the discussion of emergency powers in the future MCA, in the scenario described it would be difficult to specify how clear or imminent the risks were and it would be somewhat tenuous to describe the chronic lack of mental health beds as an emergency, especially where the shortages have come about through political choices.^17^

The situation becomes even more complex when the patient is deemed to have capacity but is still felt to be a risk, either to themselves or others. Lady Hale (past president of the Supreme Court) writes on this topic, stating:

‘But what can be done when a patient who does have capacity is about to do harm to himself? It is probably always lawful to prevent someone committing suicide . . . . Following the Human Rights Act, there is a positive duty, stemming from the right to life protected by art.2 of the ECHR, to take reasonable steps to protect the life of a detained or informal patient where there is a real and immediate risk to life about which the authorities knew or also have known at the time . . . . [However, there] was already a duty of care at common law’^11^

It should be noted that Lady Hale's comments speak to the patient's risk to themselves and not to others and if the latter risk is of concern, then the right to life in Article 2 in this circumstance may not be engaged.

Thus, an examination of the relevant law provides no definitive answer. As we have seen, the courts have tended to take the view that the MHA is comprehensive and have discouraged reliance on the MCA in similar circumstances. Furthermore, utilising the common law doctrine of necessity has been precluded by the courts, leaving us limited authority in law to continue the detention; however, it is possible that the courts’ position may change on this matter in future.^6^ We address below what our suggested next steps should be in the clinical management of the patient in our scenario.

### Reflections and considerations

#### Higher trainee perspective

The scenario described has become increasingly common to find when on call. Bed pressures and the increased demand for services mean that we are having to deal with the additional legal complexities and uncertainty relating to what to do when a section 136 is about to expire. The position in law is not always clear and one can be left between concern for safety of the patient and ensuring that one's actions remain within the bounds of the law. As a trainee one always has a more senior psychiatrist available for advice and it would be sensible that any decision is made following discussion with the on-call consultant and wider multidisciplinary team (MDT).

#### Consultant perspective

In similar scenarios, clinicians are tempted to justify their decision ‘under common law’ or ‘under the Mental Capacity Act’, without applying and documenting all principles of the relevant law or documenting the relevant principles applied to a particular case. Indeed, the use of the MCA in this scenario, when there is perceived or assessed lack of capacity, may provide a sense of reassurance to the involved clinicians about their decision being ‘legal’; however, as we have shown, any such reassurance would be false as the use of MCA in such scenarios cannot be justified. Notwithstanding all of the above, in cases where there is no demonstrable lack of capacity and section 136 powers have lapsed, an attempt to use common law to detain a patient may, in our opinion, be pushing the envelope too far. As a consultant one can be seemingly left with no viable legal options, but nevertheless, we still retain an important role in helping to manage risk within the MDT and ensuring that any actions taken can be justified on a sound ethical basis, with the patient's best interests being at the heart of any decision.

#### Legal perspective

This situation is currently a legal minefield as there is no established legal authority to extend the period of detention when a section 136 lapses. The common law doctrine of necessity would only apply in exceptional cases where there is a clear and immediate risk to life. The use of the MCA and current DoLS framework as a temporary holding power to authorise continued detention is questionable. The MCA was not designed for this purpose and should not be used to paper over cracks in the MHA legal framework. The time limit reduction for section 136 in 2017 was intended to safeguard against inappropriate and prolonged periods of detention in a place of safety. In reality however, the current crisis in mental health beds is forcing practitioners to seek alternative justifications to extend the place of safety detention until a bed becomes available. These practices are inconsistent and legally indefensible. The current situation leads to tensions in balancing the obligations to the patient's rights as laid out in the Human Rights Act 1998. The clinical team is thus placed in the unenviable position of having to balance the right to life in Article 2 with the right to liberty and ensuring lawful detention in Article 5.^18^ Furthermore, ongoing detention could arguably contravene the Article 3 right that protects from inhuman and degrading treatment. The Article 8 right could arguably be breached by ongoing detention as a violation of the patient's personal life, whereas not detaining a patient may breach the Article 8 rights of others who are entitled to have their own person protected.^18^ The clinical team is left having to consider what the least worst option is.

It is hoped that current reforms to the MHA should address this legal vacuum, focus on providing agreed national guidance in a revised MHA Code of Practice to provide clarity to practitioners and safeguard the rights of individuals detained in these circumstances.

### Practical management

For the higher trainee on call, the first port of help would always be the consultant. Although they may not be in a position to provide legal clarity, the decision about the necessity for ongoing detention should be made collaboratively and demonstrate that the senior decision maker has been involved. Legal advice can be sought – National Health Service trusts have mental health law leads and also access to advice from solicitors on an emergency basis. Further advice can also be sought from medical indemnity organisations that doctors should be members of.

Once the decision has been made to continue the detention, the legal basis on which it is being continued needs to be documented. The patient needs to be informed of this, and if their rights are being abrogated then we have a duty of candour to the patient (or if applicable their carer, advocate or family member)^19^ to let them know that this has happened. The situation needs to be flagged on trust internal systems so that it can be monitored at the board level and help inform decisions about resource availability and bed numbers, as well as contributing to the development of guidelines to assist others faced with some version of this scenario. It would be sensible for a meeting to be held after the resolution of the situation^6^ to see whether there were alternative actions that could have been taken and to help create a plan to avoid the situation in future.

In situations where the law is unclear and we are faced with ethical dilemmas, we can turn to guidance issued by the professional regulator^19^ and see whether there are answers in the bioethical frameworks within which we operate. In some situations, it may be that the patient is falsely imprisoned or unlawfully deprived of their liberty. The rationale for justifying the continued detention must be clear and start with the premise given in the General Medical Council's ‘Good medical practice’ guidance of making the care of our patients the first concern.^19^

## Conclusions

In this paper we have set out the difficulties that may arise when one finds oneself on the edges of mental health law, and where ultimately the psychiatrist has to make a decision on how to best manage the situation in an uncertain legal landscape. Although the patient in the case vignette described is fictitious, the scenario is one that is encountered by psychiatrists and gives rise to a number of ethical and legal dilemmas. We have been unable to provide a definitive answer that would be applicable in all scenarios and we reiterate calls for legislative clarity.^20^ However, we hope we have shed some further light on what has been described as a grey area, built on previous work^20^ and provided a basis for much needed discussion and debate. This is timely as we await further details of a new Mental Health Bill and an opportunity during the legislative process to seek clarity. The current uncertainty about what needs to be done serves the interests of neither patients nor the practitioners working in this arena.

## Data Availability

Data availability is not applicable to this article as no new data were created or analysed in this study.

## About the authors

**Khalil Hassanally** is a higher trainee in psychiatry in Northwick Park Hospital's Mental Health Unit, Central and North West London NHS Foundation Trust, London, UK. **Judy Laing** is Professor of Mental Health Law, Rights and Policy at the University of Bristol Law School, Bristol, UK. **Anupam Kishore** is a consultant psychiatrist on Caspian Ward at Park Royal Centre for Mental Health, Central and North West London NHS Foundation Trust, London, UK.

## References

[1] Home Office. Policing and Crime Act 2017. TSO (The Stationery Office), 2017.

[2] Crichton J. Mental Health Act 1983 – Important Changes Coming into Force on 11 December 2017. Hill Dickinson, 2017 (https://www.hilldickinson.com/insights/articles/mental-health-act-1983-important-changes-coming-force-11-december-2017 [cited 25 Oct 2021]).

[3] Cresswell M. Rise in the use of section 136 of the Mental Health Act 1983 in England and Wales: a viewpoint on Loughran. Med Sci Law 2018; 60: 140–6. 10.1177/0025802419896916 31865860

[4] Keown P, Weich S, Bhui K, Scott J. Association between provision of mental illness beds and rate of involuntary admissions in the NHS in England 1988–2008: ecological study. BMJ Clin Res 2011; 343: d3736. 10.1136/bmj.d3736 PMC3130113 21729994

[5] Fanning J. Continuities of risk in the Era of the mental capacity act. Med Law Rev 2016; 24: 415–33. 28007811 10.1093/medlaw/fww036 PMC5178325

[6] Ruck Keene A, Allen N, Butler-Cole V, Kohn N. Deprivation of Liberty in the Hospital Setting. 39 Essex Chambers, 2019. (https://1f2ca7mxjow42e65q49871m1-wpengine.netdna-ssl.com/wp-content/uploads/2018/02/Deprivation-of-liberty-in-the-hospital-setting-November-2019.pdf [cited 5 Jun 2022 Jun 5]).

[7] Department of Health. Mental Capacity Act 2005. TSO (The Stationery Office), 2005.

[8] *DN v Northumberland Tyne & Wear NHS Foundation Trust* [2011] UKUT 327 (AAC).

[9] Department of Health and Social Care. Draft MCA Code of Practice. UK Government, 2022 (https://www.gov.uk/government/consultations/changes-to-the-mca-code-of-practice-and-implementation-of-the-lps/draft-mca-code-of-practice-summary#what-is-section-4b [cited 18 Jun 2022]).

[10] Department of Health and Social Care. Draft Mental Health Bill. Department of Health and Social Care, 2022.

[11] Hale B. Mental Health Law (6th edn). Sweet and Maxwell, 2017.

[12] *Sessay, R (on the application of) v South London & Maudsley NHS Foundation Trust & Anor* [2011] EWHC 2617 (QB).

[13] *Jollah, R (On the Application of) v The Secretary of State for the Home Department* [2018] EWCA Civ 1260.

[14] 39 Essex Chambers. R (on the application of Jollah) v Secretary of State for the Home Department. 39 Essex Chambers, 2018 (https://www.39essex.com/cop_cases/r-on-the-application-of-jollah-v-secretary-of-state-for-the-home-department [cited 6 Jun 2022]).

[15] LexisNexis. Common Law Offence of False Imprisonment (Practice Notes). LexisNexis, 2017 (https://www.lexisnexis.co.uk/legal/guidance/common-law-offence-of-false-imprisonment [cited 5 Nov 2021]).

[16] *Ferreira, R (On the Application Of) v HM Senior Coroner for Inner South London* [2017] EWCA Civ 31.

[17] Cooper B. British psychiatry and its discontents. J R Soc Med 2010; 103: 397–402. 20929890 10.1258/jrsm.2010.100041 PMC2951176

[18] UK Government. Human Rights Act 1998. UK Government, 1998.

[19] General Medical Council. Good Medical Practice. GMC, 2013 (www.gmc-uk.org/guidance [cited 6 Jun 2022]).

[20] Ramesh Y, Kripalani M. Section 136: the ‘grey zone’ after the 24-h validity period lapses. BJPsych Adv 2021; 27: 62–4.

